# Xenotransplantation becoming reality

**DOI:** 10.1007/s11248-022-00306-w

**Published:** 2022-05-11

**Authors:** Konrad Fischer, Angelika Schnieke

**Affiliations:** Chair of Livestock Biotechnology, School of Life Sciences, Technical University of Munich, Munich, Germany

**Keywords:** Xenotransplantation, Organ transplantation, Pigs, Swine, Genetic engineering, Genome editing, Clinical, Bennett Senior

## Abstract

To bridge the gap between organ demand and supply, xenotransplantation has long been considered as a realistic option for end-stage organ failure. Early this year this promise became reality for David Bennett Sr., the first patient whose own failing heart was replaced with a xeno-pig heart. To get here has been a rollercoaster ride of physiological hurdles seemingly impossible to overcome, technological breakthroughs and ethical and safety concerns. It started in 1984, with Stephanie Fae Beauclair, also known as baby Fae, receiving a baboon heart, which allowed her to survive for another 30 days. For ethical reasons primate work was soon abandoned in favour of the pig. But increased phylogenetic distance also brought with it an increased immunological incompatibility. It has been the development of ever more sophisticated genetic engineering tools, which brought down the physiological barriers, enabled humanisation of porcine organs and helped addressing safety concerns. This renewed the confidence in xenotransplantation, brought new funding opportunities and resulted finally in the first in human trial.

## Introduction

According to the US government, 116,690 patients in the USA were waiting for an organ for transplant at the end of February 2022 (OPTN.Transplant 2022), indicating the need for alternatives to allotransplantation, which is the transplantation of organs between humans. Xenotransplantation, the transfer of living cells, tissues or organs between different species, has long been viewed as a feasible solution. After the initial focus on non-human primates (NHPs), the animal of choice soon became the pig. Pigs are physiologically similar to humans, reach sexual maturity within several months and have large litter sizes, have a lower risk of zoonosis than NHPs and they can be reared under specific pathogen free (SPF) housing conditions further reducing risk of infections. However, transplantation of porcine organs into humans triggers a severe immune response resulting in immediate rejection of the xenograft due to binding of preformed antibodies, activation of the complement- and coagulation system, cellular responses, inflammation and apoptosis. Consequently, porcine organs had to be genetically engineered to overcome hyperacute-, acute vascular- and cellular rejection to enable long-term graft acceptance.

## The evolving design of multi-modified xeno-pigs

Following xenotransplantation from pigs to humans, hyperacute rejection (HAR) involving complement activation, lysis of endothelial cells, vascular disruption and subsequent graft failure (Platt et al. 1991) occurs within minutes to hours. It is caused by preformed human antibodies directed against the cell surface glycosylation galactose-α1,3-galactose (α1,3-Gal). The responsible gene *GGTA1*—encoding the enzyme α1,3-galactosyltransferase—is non-functional in humans and ~ 1% of all circulating human antibodies are directed against this antigen (Galili 2005). As methods to inactivate *GGTA1* in pigs were lacking in the 1980s, the first xeno-pigs solely carried human mmcomplement regulatory transgenes (CD46, CD55 or CD59). It alone did not inhibit complement binding and together with safety concerns regarding porcine endogenous retroviruses (PERVs) dimmed the enthusiasm for xenotransplantation until in 2002 the first gene targeted pigs—with inactivation of *GGTA1—*were published (Dai et al. 2002; Lai et al. 2002).

Proving that HAR could now be overcome (Chen et al. 2005) was an essential first step for moving xenotransplantation towards the clinic. But inactivation of *GGTA1* also revealed the next hurdle acute vascular rejection (AVR), due to activation of the endothelium, complement- and coagulation system and resulting in inflammation, platelet aggregation, thrombosis and necrosis (Platt et al. 1998). But by now more tools to modify the porcine genome were also becoming available: somatic cell nuclear transfer to engineer pigs with more complex transgene arrays or to carry out gene targeting experiments. Efficiencies of the latter dramatically improved with arrival of genome editing systems especially *CRISPR/Cas9*. Non-Gal epitopes were identified (N-glycolylneuraminic acid and the SDa blood group antigen) and the responsible genes (N-acteylneuraminic acid hydroxlase, *CMAH*; β-1,4-N-galactosaminyltransferase 2, *B4GALNT2* and *B4GALNT2L* were also inactivated (Hurh et al. 2016, Byrne et al. 2018).

Inactivation of Gal and non-Gal epitopes was still not sufficient to overcome all incompatibilities, endothelium activation and cellular rejection. To alleviate these rejection responses, close to 50 different human transgenes have by now been tested in pigs. The most important ones are summarised in Table 1. With regard to cellular rejection, different approaches are being assessed e.g. inactivation or downregulation of the porcine MHC class I and class II, or expression of immunosuppressant transgenes either in a tissue-specific (Martin et al. 2005; Vabres et al. 2014) or cytokine-inducible (Fischer et al. 2020a, b, c) manner (Table 1). To adjust the size of porcine organs some groups have also inactivated the porcine growth hormone receptor (GHR) (Hinrichs et al. 2021) in commercial pig breeds, whose body weight can reach > 200 kg.

**Table 1:** Summary of gene knockouts and human transgenes, thought to be most relevant for xeno-organ transplantation. KO indicates gene inactivation

	Genes	Full gene name	Inhibition of
Incompatibilities	*GGTA1* KO	α1,3-Galactosyltransferase knockout	Hyperacute rejection (Dai et al. 2002)
	*CMAH* KO	N-acteylneuraminic acid hydroxlase knockout	Acute vascular rejection (Hurh et al. 2016)
	*B4GALNT2* KO *B4GALNT2L* KO	β-1,4-N-galactosaminyltransferase-2 knockout	Acute vascular rejection (Byrne et al. 2018)
	*GHR* KO	Growth hormone receptor knockout	Organ overgrowth (Hinrichs et al. 2021)
	*SLAI* KO	Swine leukocyte antigen class I knockout	Cross-reactive HLA antibodies (Fischer et al. 2020a, b, c)
	*hCD46*	Human Membrane cofactor protein, MCP	Complement activation (Fischer et al. 2016)
	*hCD55*	Human Decay accelerating factor, DAF	Complement activation (Fischer et al. 2016)
	*hCD59*	Human MAC-inhibitory protein	Complement activation (Fischer et al. 2016)
	*hTM, THBD*	Human Thrombomodulin	Blood coagulation (Petersen et al. 2009; Kim et al. 2015)
	*hEPCR, PROCR*	Human Endothelial Protein C receptor	Blood coagulation (Navarro et al. 2011; Iwase et al. 2014)
	*hCD39, ENTPD1*	Human Ectonucleoside triphosphate diphosphohydrolase-1	Platelet aggregation (Wheeler et al. 2012; Iwase et al. 2014)
	*hTFPI*	Human tissue factor pathway inhibitor	Blood coagulation (Iwase et al. 2014; Ji et al. 2015)
	*hA20, TNFAIP3*	Human TNF α-induced protein 3	Apoptosis and inflammation (Oropeza et al. 2009; Fischer et al. 2016)
	*hHO1, HMOX1*	Human Heme oxygenase 1	Apoptosis and inflammation (Ahrens et al. 2015; Rieblinger et al. 2018)
Cellular response	*hCD47*	Human Leukocyte surface antigen 47	Macrophages (Ide et al. 2007)
	*HLA-E/B2M*	Human Leukocyte antigen E/ Human beta 2 microglobulin	NK cells (Weiss et al. 2009)
	*CTLA4/LEA29Y*	Cytotoxic T-lymphocyte-associated antigen	T cells (Phelps et al. 2009)
	*PDL-1*	Programmed cell death ligand 1	T cells (Buermann et al. 2018)
Viral safety	*PERV* KO	Porcine endogenous retrovirus knockout	Viral transmission (Niu et al. 2017)

Increased numbers of modifications resulted in complex and very inefficient breeding strategies. To circumvent this, targeted-placement of multi-transgene constructs (Fischer et al. 2018) has now become the standard method. Genetic engineering could also help to overcome safety concerns, e.g. by generating pigs with inactivated PERVs (Niu et al. 2017), which significantly increased the confidence in the whole field. However, PERV inactivation is not a general regulatory requirement to proceed towards the clinic as transmission of PERVs has so far only been observed in in vitro co-culture experiments (Patience et al. 1997) but not in vivo.

### Towards clinical approval

**Fig. 1:**
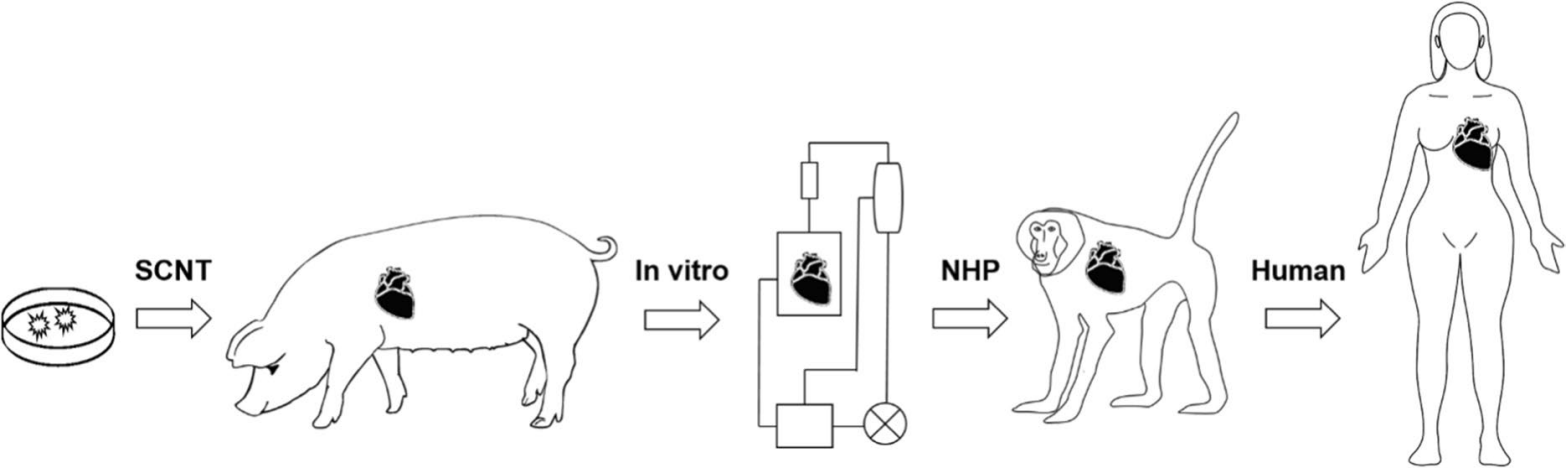
Evaluation of knockout and transgene functions. Successfully genetically engineered cells are used for somatic cell nuclear (SCNT) to generate humanised xeno-pigs. Porcine organs are assessed in vitro prior to in vivo experiments in NHP. If the outcome is promising and once all regulatory and clinical requirements are met, xeno-organs can proceed into the clinic

Multi-modified xeno-pigs can now be generated with reasonable efficiency. To show functionality and to prove that a given modification is necessary for all organs or needed for specific organs, requires both specialised in vitro assays and finally in vivo experiments generally carried out in NHPs, being the closest model animal to humans (Fig. 1).

In vitro assays can determine the effects of certain genetic modifications and thus reduce the number of animal experiments in accordance with the 3R concept (Replace, Reduce, Refine). Activation of the complement system can be assessed through incubation of porcine cells with human blood serum and subsequent detection of complement protein deposition (Fischer et al. 2016). Thrombus formation, IgM and IgG binding can be examined based on porcine endothelial cells, grown in artificial 3D vessels and perfused with human blood (Fischer et al. 2020a, b, c). Inflammation and apoptotic responses can be quantified by measurement of caspase activities, MHC class II upregulation or E-selectin levels (Fischer et al. 2016). Whole organ perfusion systems provide more detailed information on physiological parameters (Abicht et al. 2016).

Despite sophisticated in vitro assays, rejection responses and graft function have to be validated in vivo prior to clinical application. This has to be done separately for each organ type (Table 2), requiring access and permission to work with NHPs. Prior to transplantation into NHPs, mixed lymphocyte reactions, i.e. incubating porcine peripheral blood monocytes with NHP serum, can be carried out for preselection of the most suitable donor animals (Mickelson et al. 1994; Wang et al. 2020). By using an efficient immunosuppressive regimen, the survival time of some porcine transplants has now excided 1 year for heart and kidney transplantations. Survival has been even longer for islets or neurons which is less challenging than the vascularised organs. Islets can also be encapsulated to protect them from the immune system (Reichart et al. 2015; Safley et al. 2018) and are fully functional as a heterotopic transplant.

**Table 2:** Survival times of xenotransplants in NHPs

Xenograft	Genetic modification of donor pig	Survival time (days)	Reference
Islet cells	Wild type	950	Shin et al. (2016)
Neurons	*CTLA4-Ig*	549	Badin et al. (2013)
Lung	*GGTA1*KO/*CD55*/*CD47*	14	Watanabe et al. (2020)
Liver	*GGTA1*KO	29	Shah et al. (2017)
Kidney	*GGTA1*KO/*CD55*	499	Kim et al. (2019)
Heart (heterotopic)	*GGTA1*KO/*TM*/*CD46*	945	Mohiuddin et al. (2016)
Heart (orthotopic)	*GGTA1*KO/*TM*/*CD46*	195	Längin et al. (2018)

KO, indicates gene inactivation; all other genes are human transgenes

Xenotransplantation is an extremely complex undertaking with multiple factors determining the success of pre-clinical experiments. These include—besides the stability of the transgene loci or transgene expression levels in the graft—also organ storage (e.g. perfusion solutions and temperature), immunosuppression protocols, including preceding thymus transplantation to induce tolerance or pre-screening of donors by mixed lymphocyte reactions, size and age of the donor and recipient, their health status [e.g. cytomegalovirus infections can significantly reduce graft survival (Denner 2018)], blood groups, and finally the skill of the surgeon. Consequently with so many variables a comparison of results from different groups (Iwase et al. 2017; Adams et al. 2018; Kim et al. 2019) or even to pinpoint which genetic modifications or transgene expression levels are essential for organ survival is difficult.

Furthermore, although NHP are the closest human relatives with greatest immune system similarity they too have their limitations when assessing xenotransplants. Immune suppressive drugs such as anti-CD40 ligand antibodies (anti-CD154) work well in NPH but cause severe side effects in humans (Bottino et al. 2017). While *GGTA1/CMAH* double-knockout in porcine cells reduced IgG and IgM binding after incubation with human serum more efficiently than just inactivation of *GGTA1* alone, the reverse is true when using NHP serum (Estrada et al. 2015). This indicates that Neu5Gc glycosylation masks an antigen in pigs, which is recognized by (some) NHPs but not by humans. Of course, the reverse may also apply, but might only be detected after transplantation into humans.

At the end of 2021, the first xeno-kidney transplantations were performed in brain-dead humans. In the first experiment, a kidney of a *GGTA1* KO pig was simply attached to the upper leg blood vessel for 54 h. No hyperacute rejection occurred and the porcine kidney produced urine and showed normal creatinine levels (Cooper 2021). Next, two multi-modified porcine kidneys were orthotopically transplanted into a 57-year-old brain-dead human host (Porrett et al. 2022). Both kidneys were able to overcome hyperacute rejection and no intraoperative complications, measured by vascular integrity and hemodynamic stability, occurred. However, histological analysis on postoperative day 1 could detect thrombotic microangiopathy and severe tissue damage with both kidneys not being able to restore normal creatinine levels. These results may indicate a limited success, but the host was already brain-dead for 5 days prior to transplantation and died after 74 h due to multi-organ failure (Porrett et al. 2022). All such experiments require an extensive ethical review and formal approval. Still, using humans for experiments might make some people feel uncomfortable. On the other hand, humans donate organs, while here they donated their immune system to assess organ function and possibly bring lifesaving xeno-organs closer to the clinic.

## The first in patient xeno-organ transplant

The US Food and Drug Administration and other national regulatory authorities have published guidelines for xenotransplantation (EMA.EUROPA.EU 2022, FDA.GOV 2022; Liu et al. 2020). But somewhat surprisingly the first step towards clinical xenotransplantation was the approval by the FDA of meat from *GGTA1* knockout pigs to be used as a food product. The target group was people with an α-Gal allergy (red meat allergy) (Dolgin 2021). This established the requirements necessary for marketing approval of organs from genetically modified pigs.

The same group then provided the pigs for the first transplant of a porcine heart into a 57-year-old patient, David Bennett Sr., for whom no other treatment options remained. Both he himself and his family gave consent for this risky, but possibly lifesaving experiment, to go ahead. Xenotransplantation may not provide a cure, but in some cases might bridge the gap until a suitable human heart becomes available. Currently few details have been published regarding the procedure or the immune suppression regime used, except that the xeno-pigs carried 10 genetic modifications: inactivation of *GGTA1, CMAH, B4GALNT2* and *GHR* and expression of human *CD46, CD55, TM, EPCR, CD47 and HO1.* This is the same combination of genetic modifications as used for the orthotopic kidney transplant in a brain-dead patient with limited success (see above). David Bennett Sr. died almost two months after receiving the porcine graft. A detailed assessment of the precise cause of death, of the function and possible rejection of the xeno-heart is ongoing.

After more than 3 decades, has xenotransplantation finally made it into the clinic? Probably it is still too early to make definite conclusions. So far, one human patient survived for two months with a porcine heart. But hopefully this initial success will provide the confidence needed not only for the scientist and medics but especially for the patients and their families to consider xenotransplantation as a life-saving, life-extending option, or as donor organ to bridge the gap until a human donor could be found. Other organs have more complex functions compared to the heart, as such they may need their own specific combination of genetic modification to overcome species differences. Novel technologies such as single cell RNAseq or spatial single-cell transcriptomics may help to elucidate organ specific incompatibilities, which can then be addressed. The current results inspire optimism for the future of xeno-organ transplantation and it is fair to say, the future has come one step closer for xenotransplantation.

## References

[CR1] Abicht J, Mayr T, Fischer K, Reichart B, Niemann H, Panelli A, Guethoff S, Brenner P and Schnieke A (2016). Ex vivo testing of new Genetical modifications for cardiac xenotransplantation. J Heart Lung Transplant.

[CR2] Adams AB, Kim SC, Martens GR, Ladowski JM, Estrada JL, Reyes LM, Breeden C, Stephenson A, Eckhoff DE, Tector M, Tector AJ (2018). Xenoantigen deletion and chemical immunosuppression can prolong renal xenograft survival. Ann Surg.

[CR3] Ahrens HE, Petersen B, Ramackers W, Petkov S, Herrmann D, Hauschild-Quintern J, Lucas-Hahn A, Hassel P, Ziegler M, Baars W, Bergmann S, Schwinzer R, Winkler M, Niemann H (2015). Kidneys from α1,3-galactosyltransferase knockout/human heme oxygenase-1/human A20 transgenic pigs are protected from rejection during ex vivo perfusion with human blood. Transplant Direct.

[CR4] Badin RA, Vanhove B, Vadori M, Fante F, Boldrin M, DeBenedictis GM, Cavicchioli L, Sgarabotto D, Jan C and Daguin V (2013). Systemic immunosuppression plus local production of CTLA4-Ig to control rejection of transgenic pig neuroblasts in non-human primates. Joint congress of IXA and organ transplantation in ABO-incompatible and hyperimmunized recipients (IXA 2013), Wiley-Blackwell.

[CR5] Bottino R, Knoll MF, Graeme-Wilson J, Klein EC, Ayares D, Trucco M, Cooper DKC (2017) Safe use of anti-CD154 monoclonal antibody in pig islet xenotransplantation in monkeys. Xenotransplantation. 10.1111/xen.12283 10.1111/xen.12283 PMC5332295 28058735

[CR6] Buermann A, Petkov S, Petersen B, Hein R, Lucas-Hahn A, Baars W, Brinkmann A, Niemann H, Schwinzer R (2018). Pigs expressing the human inhibitory ligand PD-L1 (CD 274) provide a new source of xenogeneic cells and tissues with low immunogenic properties. Xenotransplantation.

[CR7] Byrne G, Ahmad-Villiers S, Du Z, McGregor C (2018). B4GALNT2 and xenotransplantation: a newly appreciated xenogeneic antigen. Xenotransplantation.

[CR8] Chen G, Qian H, Starzl T, Sun H, Garcia B, Wang X, Wise Y, Liu Y, Xiang Y, Copeman L, Liu W, Jevnikar A, Wall W, Cooper DK, Murase N, Dai Y, Wang W, Xiong Y, White DJ, Zhong R (2005). Acute rejection is associated with antibodies to non-Gal antigens in baboons using Gal-knockout pig kidneys. Nat Med.

[CR9] Cooper DKC (2021). Genetically engineered pig kidney transplantation in a brain-dead human subject. Xenotransplantation.

[CR10] Dai Y, Vaught TD, Boone J, Chen SH, Phelps CJ, Ball S, Monahan JA, Jobst PM, McCreath KJ, Lamborn AE, Cowell-Lucero JL, Wells KD, Colman A, Polejaeva IA, Ayares DL (2002). Targeted disruption of the alpha1,3-galactosyltransferase gene in cloned pigs. Nat Biotechnol.

[CR11] Denner J (2018). Reduction of the survival time of pig xenotransplants by porcine cytomegalovirus. Virol J.

[CR12] Dolgin E (2021). First GM pigs for allergies. Could xenotransplants be next?. Nat Biotechnol.

[CR13] EMA.EUROPA.EU (2022) https://www.ema.europa.eu/en/xenogeneic-cell-based-medicinal-products.

[CR14] Estrada JL, Martens G, Li P, Adams A, Newell KA, Ford ML, Butler JR, Sidner R, Tector M, Tector J (2015). Evaluation of human and non-human primate antibody binding to pig cells lacking GGTA1/CMAH/β4GalNT2 genes. Xenotransplantation.

[CR15] FDA.GOV (2022) https://www.fda.gov/vaccines-blood-biologics/biologics-guidances/xenotransplantation-guidances.

[CR18] Fischer K, Kraner-Scheiber S, Petersen B, Rieblinger B, Buermann A, Flisikowska T, Flisikowski K, Christan S, Edlinger M, Baars W (2016). Efficient production of multi-modified pigs for xenotransplantation by ‘combineering’, gene stacking and gene editing. Sci Rep.

[CR17] Fischer K, Kind A, Schnieke A (2018). Assembling multiple xenoprotective transgenes in pigs. Xenotransplantation.

[CR16] Fischer A, Manske K, Seissler J, Wohlleber D, Simm N, Wolf-van Buerck L, Knolle P, Schnieke A, Fischer K (2020). Cytokine-inducible promoters to drive dynamic transgene expression: the “Smart Graft” strategy. Xenotransplantation.

[CR19] Fischer K, Rieblinger B, Hein R, Sfriso R, Zuber J, Fischer A, Klinger B, Liang W, Flisikowski K, Kurome M (2020). Viable pigs after simultaneous inactivation of porcine MHC class I and three xenoreactive antigen genes GGTA1, CMAH and B4GALNT2. Xenotransplantation.

[CR20] Fischer K, Rieblinger B, Hein R, Sfriso R, Zuber J, Fischer A, Klinger B, Liang W, Flisikowski K, Kurome M, Zakhartchenko V, Kessler B, Wolf E, Rieben R, Schwinzer R, Kind A, Schnieke A (2020). Viable pigs after simultaneous inactivation of porcine MHC class I and three xenoreactive antigen genes GGTA1, CMAH and B4GALNT2. Xenotransplantation.

[CR21] Galili U (2005). The alpha-gal epitope and the anti-Gal antibody in xenotransplantation and in cancer immunotherapy. Immunol Cell Biol.

[CR22] Hinrichs A, Riedel EO, Klymiuk N, Blutke A, Kemter E, Längin M, Dahlhoff M, Keßler B, Kurome M, Zakhartchenko V, Jemiller EM, Ayares D, Bidlingmaier M, Flenkenthaler F, Hrabĕ de Angelis M, Arnold GJ, Reichart B, Fröhlich T, Wolf E (2021). Growth hormone receptor knockout to reduce the size of donor pigs for preclinical xenotransplantation studies. Xenotransplantation.

[CR23] Hurh S, Kang B, Choi I, Cho B, Lee EM, Kim H, Kim YJ, Chung YS, Jeong JC, Hwang JI, Kim JY, Lee BC, Surh CD, Yang J, Ahn C (2016). Human antibody reactivity against xenogeneic N-glycolylneuraminic acid and galactose-α-1,3-galactose antigen. Xenotransplantation.

[CR24] Ide K, Wang H, Tahara H, Liu J, Wang X, Asahara T, Sykes M, Yang YG, Ohdan H (2007). Role for CD47-SIRPalpha signaling in xenograft rejection by macrophages. Proc Natl Acad Sci USA.

[CR25] Iwase H, Ezzelarab MB, Ekser B, Cooper DK (2014). The role of platelets in coagulation dysfunction in xenotransplantation, and therapeutic options. Xenotransplantation.

[CR26] Iwase H, Hara H, Ezzelarab M, Li T, Zhang Z, Gao B, Liu H, Long C, Wang Y, Cassano A, Klein E, Phelps C, Ayares D, Humar A, Wijkstrom M and Cooper DKC (2017) Immunological and physiological observations in baboons with life-supporting genetically engineered pig kidney grafts. Xenotransplantation. 10.1111/xen.12293. 10.1111/xen.12293 PMC5397334 28303661

[CR27] Ji H, Li X, Yue S, Li J, Chen H, Zhang Z, Ma B, Wang J, Pu M, Zhou L, Feng C, Wang D, Duan J, Pan D, Tao K, Dou K (2015). Pig BMSCs transfected with human TFPI combat species incompatibility and regulate the human TF pathway in vitro and in a rodent model. Cell Physiol Biochem.

[CR28] Kim H, Hawthorne WJ, Kang HJ, Lee YJ, Hwang JI, Hurh S, Ro H, Jeong JC, Cho B, Yang J, Ahn C (2015). Human thrombomodulin regulates complement activation as well as the coagulation cascade in xeno-immune response. Xenotransplantation.

[CR29] Kim SC, Mathews DV, Breeden CP, Higginbotham LB, Ladowski J, Martens G, Stephenson A, Farris AB, Strobert EA, Jenkins J, Walters EM, Larsen CP, Tector M, Tector AJ, Adams AB (2019). Long-term survival of pig-to-rhesus macaque renal xenografts is dependent on CD4 T cell depletion. Am J Transplant.

[CR30] Lai L, Kolber-Simonds D, Park K-W, Cheong H-T, Greenstein JL, Im G-S, Samuel M, Bonk A, Rieke A, Day BN, Murphy CN, Carter DB, Hawley RJ, Prather RS (2002). Production of α-1,3-galactosyltransferase knockout pigs by nuclear transfer cloning. Science.

[CR31] Längin M, Mayr T, Reichart B, Michel S, Buchholz S, Guethoff S, Dashkevich A, Baehr A, Egerer S, Bauer A, Mihalj M, Panelli A, Issl L, Ying J, Fresch AK, Buttgereit I, Mokelke M, Radan J, Werner F, Lutzmann I, Steen S, Sjöberg T, Paskevicius A, Qiuming L, Sfriso R, Rieben R, Dahlhoff M, Kessler B, Kemter E, Kurome M, Zakhartchenko V, Klett K, Hinkel R, Kupatt C, Falkenau A, Reu S, Ellgass R, Herzog R, Binder U, Wich G, Skerra A, Ayares D, Kind A, Schönmann U, Kaup F-J, Hagl C, Wolf E, Klymiuk N, Brenner P and Abicht J-M (2018). Consistent success in life-supporting porcine cardiac xenotransplantation. Nature.

[CR32] Liu Y, Qin L, Tong R, Liu T, Ling C, Lei T, Zhang D, Wang Y, Deng S (2020). Regulatory changes in China on xenotransplantation and related products. Xenotransplantation.

[CR33] Martin C, Plat M, Nerriére-Daguin V, Coulon F, Uzbekova S, Venturi E, Condé F, Hermel JM, Hantraye P, Tesson L, Anegon I, Melchior B, Peschanski M, Le Mauff B, Boeffard F, Sergent-Tanguy S, Neveu I, Naveilhan P, Soulillou JP, Terqui M, Brachet P, Vanhove B (2005). Transgenic expression of CTLA4-Ig by fetal pig neurons for xenotransplantation. Transgenic Res.

[CR34] Mickelson EM, Guthrie LA, Etzioni R, Anasetti C, Martin PJ, Hansen JA (1994). Role of the mixed lymphocyte culture (MLC) reaction in marrow donor selection: matching for transplants from related haploidentical donors. Tissue Antigens.

[CR35] Mohiuddin MM, Singh AK, Corcoran PC, Thomas Iii ML, Clark T, Lewis BG, Hoyt RF, Eckhaus M, Iii RNP, Belli AJ, Wolf E, Klymiuk N, Phelps C, Reimann KA, Ayares D and Horvath KA (2016) Chimeric 2C10R4 anti-CD40 antibody therapy is critical for long-term survival of GTKO.hCD46.hTBM pig-to-primate cardiac xenograft. Nat Commun 7: 11138. 10.1038/ncomms11138. 10.1038/ncomms11138 PMC4822024 27045379

[CR36] Navarro S, Bonet E, Estellés A, Montes R, Hermida J, Martos L, España F, Medina P (2011). The endothelial cell protein C receptor: its role in thrombosis. Thromb Res.

[CR37] Niu D, Wei HJ, Lin L, George H, Wang T, Lee IH, Zhao HY, Wang Y, Kan Y, Shrock E, Lesha E, Wang G, Luo Y, Qing Y, Jiao D, Zhao H, Zhou X, Wang S, Wei H, Guell M, Church GM, Yang L (2017). Inactivation of porcine endogenous retrovirus in pigs using CRISPR-Cas9. Science.

[CR38] OPTN.Transplant (2022) https://optn.transplant.hrsa.gov/data/view-data-reports/national-data/.

[CR39] Oropeza M, Petersen B, Carnwath JW, Lucas-Hahn A, Lemme E, Hassel P, Herrmann D, Barg-Kues B, Holler S, Queisser AL, Schwinzer R, Hinkel R, Kupatt C, Niemann H (2009). Transgenic expression of the human A20 gene in cloned pigs provides protection against apoptotic and inflammatory stimuli. Xenotransplantation.

[CR40] Patience C, Takeuchi Y, Weiss RA (1997). Infection of human cells by an endogenous retrovirus of pigs. Nat Med.

[CR41] Petersen B, Ramackers W, Tiede A, Lucas-Hahn A, Herrmann D, Barg-Kues B, Schuettler W, Friedrich L, Schwinzer R, Winkler M, Niemann H (2009). Pigs transgenic for human thrombomodulin have elevated production of activated protein C. Xenotransplantation.

[CR42] Phelps CJ, Ball SF, Vaught TD, Vance AM, Mendicino M, Monahan JA, Walters AH, Wells KD, Dandro AS, Ramsoondar JJ, Cooper DK, Ayares DL (2009). Production and characterization of transgenic pigs expressing porcine CTLA4-Ig. Xenotransplantation.

[CR43] Platt JL, Fischel RJ, Matas AJ, Reif SA, Bolman RM, Bach FH (1991). Immunopathology of hyperacute xenograft rejection in a swine-to-primate model. Transplantation.

[CR44] Platt JL, Lin SS, McGregor CG (1998). Acute vascular rejection. Xenotransplantation.

[CR45] Porrett PM, Orandi BJ, Kumar V, Houp J, Anderson D, Cozette Killian A, Hauptfeld-Dolejsek V, Martin DE, Macedon S, Budd N, Stegner KL, Dandro A, Kokkinaki M, Kuravi KV, Reed RD, Fatima H, Killian JT, Baker G, Perry J, Wright ED, Cheung MD, Erman EN, Kraebber K, Gamblin T, Guy L, George JF, Ayares D, Locke JE (2022). First clinical-grade porcine kidney xenotransplant using a human decedent model. Am J Transplant.

[CR46] Reichart B, Niemann H, Chavakis T, Denner J, Jaeckel E, Ludwig B, Marckmann G, Schnieke A, Schwinzer R, Seissler J, Tönjes RR, Klymiuk N, Wolf E, Bornstein SR (2015). Xenotransplantation of porcine islet cells as a potential option for the treatment of type 1 diabetes in the future. Horm Metab Res.

[CR47] Rieblinger B, Fischer K, Kind A, Saller BS, Baars W, Schuster M, Wolf-van Buerck L, Schäffler A, Flisikowska T, Kurome M, Zakhartchenko V, Kessler B, Flisikowski K, Wolf E, Seissler J, Schwinzer R, Schnieke A (2018). Strong xenoprotective function by single-copy transgenes placed sequentially at a permissive locus. Xenotransplantation.

[CR48] Safley SA, Kenyon NS, Berman DM, Barber GF, Willman M, Duncanson S, Iwakoshi N, Holdcraft R, Gazda L, Thompson P, Badell IR, Sambanis A, Ricordi C, Weber CJ (2018). Microencapsulated adult porcine islets transplanted intraperitoneally in streptozotocin-diabetic non-human primates. Xenotransplantation.

[CR49] Shah JA, Patel MS, Elias N, Navarro-Alvarez N, Rosales I, Wilkinson RA, Louras NJ, Hertl M, Fishman JA, Colvin RB, Cosimi AB, Markmann JF, Sachs DH, Vagefi PA (2017). Prolonged survival following pig-to-primate liver xenotransplantation utilizing exogenous coagulation factors and costimulation blockade. Am J Transplant.

[CR50] Shin JS, Min BH, Kim JM, Kim JS, Yoon IH, Kim HJ, Kim YH, Jang JY, Kang HJ, Lim DG, Ha J, Kim SJ, Park CG (2016). Failure of transplantation tolerance induction by autologous regulatory T cells in the pig-to-non-human primate islet xenotransplantation model. Xenotransplantation.

[CR51] Vabres B, Le Bas-Bernardet S, Riochet D, Cherel Y, Minault D, Hervouet J, Ducournau Y, Moreau A, Daguin V, Coulon F, Pallier A, Brouard S, Robson SC, Nottle MB, Cowan PJ, Venturi E, Mermillod P, Brachet P, Galli C, Lagutina I, Duchi R, Bach JM, Blancho G, Soulillou JP, Vanhove B (2014). hCTLA4-Ig transgene expression in keratocytes modulates rejection of corneal xenografts in a pig to non-human primate anterior lamellar keratoplasty model. Xenotransplantation.

[CR52] Wang ZY, Morsi M, Nguyen HQ, Bikhet M, Burnette K, Ayares D, Cooper DKC, Hara H (2020). The human T-cell proliferative response to triple-knockout pig cells in mixed lymphocyte reaction. Xenotransplantation.

[CR53] Watanabe H, Ariyoshi Y, Pomposelli T, Takeuchi K, Ekanayake-Alper DK, Boyd LK, Arn SJ, Sahara H, Shimizu A, Ayares D, Lorber MI, Sykes M, Sachs DH, Yamada K (2020). Intra-bone bone marrow transplantation from hCD47 transgenic pigs to baboons prolongs chimerism to >60 days and promotes increased porcine lung transplant survival. Xenotransplantation.

[CR54] Weiss EH, Lilienfeld BG, Müller S, Müller E, Herbach N, Kessler B, Wanke R, Schwinzer R, Seebach JD, Wolf E, Brem G (2009). HLA-E/human beta2-microglobulin transgenic pigs: protection against xenogeneic human anti-pig natural killer cell cytotoxicity. Transplantation.

[CR55] Wheeler DG, Joseph ME, Mahamud SD, Aurand WL, Mohler PJ, Pompili VJ, Dwyer KM, Nottle MB, Harrison SJ, d'Apice AJ, Robson SC, Cowan PJ, Gumina RJ (2012). Transgenic swine: expression of human CD39 protects against myocardial injury. J Mol Cell Cardiol.

